# Determinants of breastfeeding initiation among mothers in Kuwait

**DOI:** 10.1186/1746-4358-5-7

**Published:** 2010-07-28

**Authors:** Manal Dashti, Jane A Scott, Christine A Edwards, Mona Al-Sughayer

**Affiliations:** 1 Human Nutrition Section, Division of Developmental Medicine, University of Glasgow, Glasgow, UK; 2 Nutrition and Dietetics, School of Medicine, Flinders University, Adelaide, Australia; 3 Department of Biological Sciences, Faculty of Science, Kuwait University, Kuwait

## Abstract

**Background:**

Exclusive breastfeeding is recommended as the optimal way to feed infants for the first six months of life. While overall breastfeeding rates are high, exclusive breastfeeding is relatively uncommon among Middle Eastern women. The objective of this study was to identify the incidence of breastfeeding amongst women in the six governorates of Kuwait and the factors associated with the initiation of breastfeeding.

**Methods:**

A sample of 373 women (aged 17-47 years), recruited shortly after delivery from four hospitals in Kuwait, completed a structured, interviewer-administered questionnaire. Multivariate logistic regression analysis was used to identify those factors independently associated with the initiation of breastfeeding.

**Results:**

In total, 92.5% of mothers initiated breastfeeding and at discharge from hospital the majority of mothers were partially breastfeeding (55%), with only 30% of mothers fully breastfeeding. Prelacteal feeding was the norm (81.8%) and less than 1 in 5 infants (18.2%) received colostrum as their first feed. Only 10.5% of infants had been exclusively breastfed since birth, the remainder of the breastfed infants having received either prelacteal or supplementary infant formula feeds at some time during their hospital stay. Of the mothers who attempted to breastfeed, the majority of women (55.4%) delayed their first attempt to breastfeed until 24 hours or more after delivery. Breastfeeding at discharge from hospital was positively associated with paternal support for breastfeeding and negatively associated with delivery by caesarean section and with the infant having spent time in the Special Care Nursery.

**Conclusions:**

The reasons for the high use of prelacteal and supplementary formula feeding warrant investigation. Hospital policies and staff training are needed to promote the early initiation of breastfeeding and to discourage the unnecessary use of infant formula in hospital, in order to support the establishment of exclusive breastfeeding by mothers in Kuwait.

## Background

There is an ever increasing volume of evidence highlighting the importance of breastfeeding in infancy and later life. International recommendations promote exclusive breastfeeding as the optimal method of infant feeding for the first six months of life [1]. Studying breastfeeding practices in women is important to identify those population groups most likely to not breastfeed and to identify and understand their reasons for not breastfeeding. The identification of the determinants of breastfeeding practices will inform the design of targeted interventions to promote breastfeeding [2] and the formulation of national public health policy.

Kuwait is located in the Middle East, bordering the Persian Gulf, between Iraq and Saudi Arabia. Less than one half of the population are Kuwaiti nationals (45%) with the other predominant ethnic groups being other Arabic groups (35%), South Asian (9%), Iranian (4%) and other nationalities (7%). Arabic is the official language, while English is widely spoken [3]. Despite the large amount of evidence that breastfeeding reduces the risk of diseases in infancy and later life, efforts to promote breastfeeding have been limited and irregular in Kuwait. Breastfeeding practices have not been well studied in Kuwait with no major studies having been conducted since the late 1980s when a large cross sectional survey conducted in 1989 reported an initiation rate of any breastfeeding of 86% and exclusive breastfeeding of 60.6% [4]. The proportion of children breastfeeding at six months in Kuwait is well below international targets ranging from 35% to 44% [5,6].

The purpose of the Kuwait Infant Feeding Study (KIFS) is to identify the incidence and prevalence of breastfeeding up to 26 weeks postpartum among a population of women living in Kuwait and to identify the factors associated with the initiation and duration of breastfeeding. Data collected in this study will contribute to the limited breastfeeding surveillance data available for Kuwait and inform future public health policy. The aim of this paper is to present the results of the cross-sectional analysis of the baseline data related to the initiation of breastfeeding and infant feeding practices prior to discharge from hospital.

## Methods

A longitudinal study of infant feeding patterns among women in Kuwait was conducted over the period of October 2007 to October 2008. Mothers were recruited from three main governmental hospitals (Maternity, Al-Addan and Al Farwania), located in different areas of Kuwait, as well as one private hospital located in the Bnaid al Gar area which services patients from various areas across Kuwait. Within 72 hours of delivery, mothers were visited on the postnatal ward and given verbal and written information explaining the aims of the study and what their participation would involve. The questionnaire was administered by the principal researcher (MD) via a 30 minute face-to-face interview, which had the advantage of ensuring that the questionnaire was fully completed. The study aimed to recruit approximately 500 mothers over a six months period and a weekly recruitment target of 20 mothers per week was set for logistical reasons, as the principal researcher was the sole person responsible for recruiting subjects and for conducting the subsequent follow-up telephone interviews at 6, 12, 18 and 26 weeks postpartum. Data from these surveys will be analyzed and reported separately.

### Data collection

The structured baseline questionnaire used in this study was adapted from the first Perth Infant Feeding Study (PIFS) [7] and was designed to identify feeding practices while in hospital and to collect information on variables known or suspected to be associated with breastfeeding initiation, including socio-demographic, biomedical and psychosocial factors, and hospital practices. The PIFS questionnaire has been shown to have good content validity and has been used in other Australian studies [8] and translated from English into a variety of languages for use in similar studies of infant feeding practices in China and Kenya [9,10]. The PIFS questionnaire was modified slightly for use in this study to suit the various cultural differences of women living in Kuwait, for example the questions on marital status and alcohol intake were removed. It was translated from English into Arabic by the principal researcher and back into English by a second person unfamiliar with the subject matter to ensure the face validity of the questionnaire was retained. The Arabic version was pilot-tested on a group of 25 Arabic speaking new mothers and minor modifications were made based on some of their comments and suggestions.

Breastfeeding terms and definitions used in this study are those internationally recommended by the World Health Organization. Full breastfeeding is breastfeeding either exclusively or predominantly and exclusive breastfeeding means giving a baby no other food or drink, including water, in addition to breast milk (medicines and vitamin and minerals drops are permitted) [11]. Ever breastfed includes all infants who were put to the breast on at least one occasion. Breastfeeding at discharge was defined as the method of feeding at the time that the baseline questionnaire was completed.

### Statistical analysis

Data were entered and analyzed using the Statistical Package for Social Sciences (SPSS, version 16.0) [12]. As a preliminary investigation of the data, contingency tables of feeding method versus explanatory factors were made and univariate logistic regression analysis was performed. Multivariate logistic regression analysis was employed to determine which individual variables were independently associated with the initiation of breastfeeding. All variables reported in the literature to be associated with the decision to breastfeed and investigated in this study were included in the full model which was reduced using the backward stepwise procedure. All variables in the final model were variables for which when excluded, the change in deviance compared with the corresponding chi-square test statistic on the relevant degrees of freedom was significant.

### Ethical considerations

Signed informed consent was obtained from all participants who were advised that they could withdraw from the study without having to provide justification or affecting their hospital care. The confidentiality of the data and the privacy of mothers were respected at all times. The project received ethical approval from the University of Glasgow Medical Faculty Ethics Committee, the Faculty of Medicine at Kuwait University and the Ministry of Health in Kuwait. Letters of approval were obtained from all participating hospitals.

## Results

A total of 439 women were invited to participate in the study and 373 mothers completed the baseline questionnaire while in hospital, giving a response rate of 85%. Table 1 describes the socio-demographic and biomedical characteristics of participants. The age of women ranged from 17-47 years, with an average of 29.16 years (SD = 6.6 years), and most women had 12 or more years of schooling (66%).

**Table 1:** Socio-demographic and biomedical characteristics of participants (n = 373)

Maternal characteristics	N	%
Maternal age		
< 25 yrs	82	22.0
25-34 yrs	240	64.3
≥35 yrs	51	13.7
Maternal education		
< 12 yrs	127	34.0
≥ 12 yrs	246	66.0
Country of mother's birth		
Kuwait	196	52.5
Other Gulf States	13	3.5
Other Arabian countries	123	33.0
Other Islamic countries	10	2.7
Other world countries	31	8.3
Mother's living area (Kuwaiti governorates)		
Hawalli	186	49.9
Farwaniya	96	25.7
Mubarak al Kabeer & Ahmadi	49	13.1
Kuwait City & Jahraa	42	11.3
Father's occupation		
Managers & professionals	97	26.0
Sales & clerical	115	30.8
Unskilled occupations	157	42.1
Unemployed	4	1.1
Employment plans for the next 6 months		
Stay at home with the baby	207	55.5
Work full time	20	5.4
Work part time	113	30.3
Study full time	7	1.9
Study part time	6	1.6
Undecided	20	5.4
Parity		
Primiparous	112	30.0
Multiparous	261	70.0
Vaginal delivery		
Yes	235	63.0
No	138	37.0
**Infant characteristics**		
Gender		
Male	195	52.3
Female	178	47.7
Spent time in SCN		
Yes	76	20.4
No	297	79.6

All of the mothers declining to participate in the study (n = 66) were asked three short questions related to their socio-demographic status and chosen method of feeding to allow comparison with the study sample. There were no significant differences between participants and those declining to participate with respect to age (χ² 4.413, *P *= 0.110), level of education (χ² 2.455, *P *= 0.117) and chosen method of feeding at discharge (χ² 447, *P *= 0.800), suggesting that the sample was representative of the population from which it was drawn. However, just over one third of women (37%) had delivered by caesarean section, which was significantly higher than the 11.5% reported for Kuwaiti women in general [13]

In total, 92.5% of mothers initiated breastfeeding however, at discharge from hospital, 84.8% of participants were breastfeeding their infants with less than one third of mothers (29.8%) fully breastfeeding their infants (Table 2). Prelacteal feeding was the norm, (81.8%) with less than 1 in 5 infants (18.2%) receiving colostrum as their first feed. Only 10.5% of infants had been exclusively breastfed since birth, the remainder of breastfed infants having received prelacteal feeds of either infant formula (76.4%) or glucose water (4.6%) and/or supplementary feeds of infant formula at some time during their hospital stay.

**Table 2:** Infant feeding practices

Feeding practices	*n*	%	95% CI
Initiated breastfeeding^a^	345	92.5	89.8, 95.2
Feeding method at discharge			
Fully breastfed	111	29.8	25.2, 34.4
*(Exclusively breastfed since birth)*	*(39)*	*(10.5)*	*(7.4, 13.6)*
Partially breastfed	205	55.0	50.0, 60.0
Fully formula fed	57	15.3	11.6, 19.0
Received prelacteal feed	305	81.8	77.0, 85.0

^a ^Includes those infants who were ever breastfed on at least one occasion

Of the mothers who attempted to breastfeed in hospital, 24.3% first put their baby to the breast within 6 hours of delivery, 20.4% between 6 and 24 hours, with the majority of women (55.3%) delaying their first attempt to breastfeed until 24 hours or more after delivery.

### Univariate analysis

Table 3 lists a variety of socio-demographic, biomedical and psychosocial factors that might be expected to have an influence on breastfeeding initiation at discharge. The univariate odds ratios indicate the likelihood of a mother breastfeeding at discharge from hospital. In this study, no association was found between breastfeeding at discharge and any of the socio-demographic factors including maternal age, education, employment and country of origin. Among the biomedical factors, no association was observed with parity, but there was a negative association between mode of delivery and breastfeeding at discharge. Women who had a caesarean section delivery were significantly less likely to be breastfeeding at discharge than women who had delivered vaginally (OR 0.55, 95% CI 0.31, 0.98). Also, infants who had not been admitted to the Special Care Nursery (SCN) for short-term observation after delivery were significantly more likely to be breastfed (OR 2.77, 95% CI 1.50, 5.10) than those admitted to the SCN. No association was found with any of the psychosocial factors investigated.

**Table 3:** Number (percentage) and univariate odds ratios (95% confidence intervals) for any breastfeeding at discharge from hospital (n = 373)

Variables	Any breastfeeding at discharge		Univariate odds ratio	
	Yes	No		
	N (%)	N (%)	OR	95% CI
**Sociodemographic**				
Mother's age (years):				
< 25	71 (86.6)	11 (13.4)	1.00	
25-34	204 (85.0)	36 (15.0)	1.57	0.62, 4.02
≥ 35	41 (80.4)	10 (19.6)	1.38	0.64, 3.00
Maternal Education (years of schooling):				
< 12	106 (83.5)	21 (16.5)	1.00	
≥12	210 (85.4)	36 (14.6)	1.60	0.64, 2.08
Mother's country of birth:				
Kuwait & Gulf States	172 (82.3)	37 (17.7)	1.00	
Other Arabic countries	109 (88.6)	14 (11.4)	1.67	0.87, 3.24
Other world countries	35 (85.4)	6 (14.6)	1.25	0.49, 3.20
Mother employed/studying part- or full-time at 6 months before birth				
Yes	132 (82.5)	28 (17.5)	1.00	
No	184 (86.4)	29 (13.6)	1.35	0.76, 2.37
Mother intended to be employed/studying part- or full time at 6 months postpartum				
Yes	119 (81.5)	27 (18.5)	1.00	
No	179 (86.5)	28 (13.5)	0.69	0.39, 1.23
Don't know yet/undecided	18 (90.0)	2 (10.0)	0.31	0.31, 6.40
Mother's occupation:				
Managers & professionals	83 (88.3)	11 (11.7)	1.00	
Sales & clericals	62 (82.7)	13 (17.3)	0.63	0.26, 1.50
Unskilled occupations	26 (81.2)	6 (18.8)	0.57	0.19, 1.70
House wives	145 (84.3)	27 (15.7)	0.71	0.34, 1.51
Father's occupation:				
Managers & professionals	85 (87.6)	12 (12.4)	1.00	
Sales & clericals	93 (80.9)	22 (19.1)	2.36	0.23, 24.58
Unskilled occupations	135 (75)	22 (14)	1.41	0.14, 14.2
Unemployed	3 (75)	1 (25)	2.04	0.20, 20.56
Location:				
Kuwait City & Jahraa	31 (73.8)	11 (26.2)	1.00	
Hawalli	163 (87.6)	23 (12.4)	2.51	1.11, 5.68
Farwania	80 (83.3)	16 (16.7)	1.77	0.74, 4.24
Mubarak Al Kabeer & Ahmedi	42 (85.7)	7 (14.3)	2.13	0.74, 6.12
**Biomedical** Parity:				
Primiparous	92 (82.1)	20 (17.9)	1.00	
Multiparous	224 (85.8)	37 (14.2)	1.32	0.72, 2.39
Vaginal Delivery:				
Yes	206 (87.7)	29 (12.3)	1.00	
No	110 (79.7)	28 (20.3)	0.55	0.31, 0.98
Infant admitted to special care nursery:				
Yes	55 (72.4)	21 (27.6)	1.00	
No	261 (87.9)	36 (12.1)	2.77	1.50, 5.10
**Psychosocial**				
Mother attended antenatal classes for this or previous pregnancy:				
Yes	29 (93.5)	2 (6.5)	1.00	
No	287 (83.9)	55 (16.1)	0.36	0.08, 1.55
Father prefers breastfeeding				
Yes	259 (86.3)	41 (13.7)	1.00	
No or ambivalent	57 (78.1)	16 (21.9)	0.56	0.30, 1.07
Maternal grandmother prefers breastfeeding				
Yes	286 (84.6)	52 (15.4)	1.00	
No or ambivalent	30 (85.7)	5 (14.3)	1.09	0.40, 2.94
Maternal grandmother breastfed at least one infant				
Yes	297 (84.1)	56 (15.9)	1.00	
No or don't know	19 (95.0)	1 (5.0)	0.28	0.04, 2.13
Infant feeding decision made before pregnancy				
Yes	241 (84.3)	45 (15.7)	1.00	
No	75 (86.2)	12 (13.8)	1.17	0.59, 2.32

### Multivariate analysis

The understanding of the factors associated with the initiation of breastfeeding was enhanced by modeling all of the factors identified in table 3 using multivariate logistic regression. Those factors that were independently associated with initiating breastfeeding and breastfeeding at discharge (both any and exclusive breastfeeding) are presented in Table 4. After potential confounding factors were controlled for, infants who were delivered by caesarean section were less likely to be exclusively breastfed at discharge from hospital (adjOR 0.15: 95% CI 0.05, 0.43). Mothers whose infants had not been admitted to SCN were significantly more likely to have initiated breastfeeding (adjOR 5.67: 95% CI 2.49, 12.95) and to be exclusively breastfeeding (adjOR 4.23: 95% CI 0.98, 18.34) or feeding their infants any breast milk at discharge from hospital (adjOR 2.85: 95% CI 1.52, 5.33). Women who perceived that their husband either preferred formula feeding or was ambivalent about how she would feed their infant were less likely to be breastfeeding at discharge than those whose husbands preferred breastfeeding (adjOR 0.49: 95% CI 0.25, 0.96). Mothers originally from other Arab countries were found to be more likely to initiate breastfeeding (adjOR 3.47: 95% CI 1.12, 10.80) and to be exclusively breastfeeding at discharge (adjOR 3.12: 95% CI 1.46, 6.66) than mothers from Kuwait and other Gulf States.

**Table 4:** Factors independently^a ^associated with initiation of breastfeeding and any and exclusive breastfeeding at discharge from hospital after adjustment for potential confounders^b ^(n = 373)

Variables	*n*	Ever initiated breastfeeding		Any breastfeeding at discharge from hospital		Exclusive breastfeeding at discharge from hospital	
		AdjOR^c^	CI 95%	AdjOR	CI 95%	AdjOR	CI 95%
**Biomedical**							
Method of delivery							
Vaginal (ref)	235	NS		1.00		1.00	
Cesarean section	138			0.60	0.33, 1.06	0.15	0.05, 0.43
Infant admitted to SCU							
Yes (ref)	76	1.00		1.00		1.00	
No	297	5.67	2.49, 12.95	2.85	1.52, 5.33	4.23	0.98, 18.34
**Psychosocial**							
Father prefers breastfeeding							
Yes (ref)	300	NS		1.00		NS	
No or ambivalent	73			0.49	0.25, 0.96		
**Sociodemographic**							
Country of mother's birth							
Kuwait & Gulf countries (ref)	209	1.00		NS		1.00	
Other Arab countries	123	3.47	1.12, 10.80			3.12	1.46, 6.66
Other world countries	41	1.38	0.38, 8.82			1.49	0.45, 4.93

^a^All variables in the final model were variables for which, when excluded, the change in deviance compared with the corresponding χ^2 ^test statistic on the relevant degrees of freedom was significant.

^b ^Non-significant variables were maternal grandmother preference of breastfeeding, when the feeding decision was made, previous employment status, future employment intentions, number of years schooling, whether maternal grandmother breastfed any of her children, father's occupation, parity, maternal age, mother's occupation, attendance at antenatal classes

^c^AdjOR: Adjusted Odds Ratio

## Discussion

The initiation rate of 92.5% (95% CI 89.8, 95.2) reported in our study was significantly higher than the rate of 86% (95% CI 84.7, 87.3) reported for Kuwait in 1989 [4] and comparable to recent breastfeeding initiation rates for other Middle Eastern countries, which are reportedly 100% in Iran [14], 95.4% in Egypt [15], 89.1% to 98.2% in Turkey [16,17], 95.4% in Lebanon [18], 91.3% to 95% in Iraq [19,20], and 91.1% in Tunisia [15]. Nevertheless, while this study revealed that the majority of women in Kuwait initiated breastfeeding, less than one-third of infants were fully breastfed and the use of prelacteal feeds was very common. Subsequently only one in ten infants had been exclusively breastfed since birth.

There is evidence that the implementation of the Baby Friendly Hospital Initiative (BFHI) in maternity wards improved breastfeeding practices [21-23]. None of the hospitals in this study were BFHI accredited at the time of the study and it is clear, from the results of this study, that two of the BFHI Ten Steps to Successful Breastfeeding that are; "*Help mothers initiate breastfeeding within half an hour of birth*" and "*Give newborn infants no food or drink other than breast milk, unless medically indicated"*, were not practiced in the participating hospitals. In this study, the majority of women had not attempted to breastfeed until 24 or more hours after birth, contributing to the high incidence of prelacteal feeding.

The practice of delayed breastfeeding initiation deprives infants of the benefits of colostrum [24] and delaying initiation beyond two hours postpartum has been associated with shorter breastfeeding duration [25]. The practice has been reported in other Middle Eastern countries, for instance, only 6% of mothers in an Iranian study [26] breastfed within five hours of delivery, while in an Egyptian study [27] most women (71.6%) gave the first breastfeed more than 36 hours after delivery. Similarly, only 10% of Turkish mothers breastfed their infants within the first hour of birth, with most women (90%) initiating breastfeeding two days after birth [28]. As a consequence, high rates of prelacteal feeding have been reported among populations of Middle Eastern women, with lower rates being reported in a Lebanese study [18] where 49%of women offered prelacteal feeds, 61.0% in a Jordanian survey [29] and 60.2% in an Iraqi study [30]. Prelacteal feeding was almost universal in an Iranian study [31] where 96.1% of mothers gave sugar water as the first feed after birth and reported rates of prelacteal feeding in Egypt ranged from 48.8% [32] to as high as 97.6% [27]. In addition to prelacteal feeding, mixed feeding during the hospital stay was common in our study and is a practice which has consistently been shown to be negatively associated with the initiation of breastfeeding in Egypt [33-35] and the duration of exclusive breastfeeding in Turkey [36], Saudi Arabia [37,38] and Western countries [39-41].

High rates of delayed breastfeeding initiation and prelacteal feeding in various Muslim cultures are related to the traditional beliefs held by women that colostrum should not be fed to the infant because it is of limited nutritional value or because it might harm the infant [42]. For instance, Pakistani [43], Somali [44] and Turkish [28] women reportedly believe colostrum to be dirty, stale milk that has been stored in the breast for nine months. Similarly, Gambian women [45] believe that colostrum is "hot milk" which could give their baby stomach ache and diarrhoea, while some Pakistani women [43] believe that colostrum might even kill their infant. These beliefs, while more common in less literate women [28], are firmly entrenched and reinforced by religious leaders [28] and elders, both female and male [45] and supported by traditional birthing assistants [45].

As we did not expressly ask women about their beliefs related to the value of colostrum, we do not know if this negative view is prevalent amongst Kuwaiti women and can help explain the high rates of delayed breastfeeding and prelacteal feeding observed. The reasons for the high prevalence of these practices amongst Kuwaiti and the associated beliefs and attitudes of mothers, grandmothers and health professionals warrant further investigation and are probably best studied using qualitative research methodologies which are better suited to eliciting information on sensitive issues than the quantitative methodology employed in this study.

The current study failed to find an association between breastfeeding initiation or prevalence at discharge and a variety of socio-demographic factors that have been reported to be associated with breastfeeding initiation in other studies of Middle Eastern women. For instance, no positive association was found between maternal age and breastfeeding initiation previously reported in an earlier study in Kuwait [5] and in other studies of Middle Eastern women in the United Arab Emirates (UAE) [46,47] and Saudi Arabia [48]. Similarly, no association was found for breastfeeding initiation with level of maternal education, whereas an earlier study in Kuwait [5] and studies conducted in Lebanon [49], Saudi Arabia [50] and Qatar [51] all reported an inverse association between maternal level of education and the initiation of breastfeeding. Conversely, a study in Egypt reported that educated mothers were more likely to initiate breastfeeding earlier and to exclusively breastfeed their infants in the first week of life than less educated women [32], which is consistent with most studies from Western countries [52].

Of interest in this study was the finding that non-Kuwaiti mothers who were from other Arab countries were more likely than Kuwaiti born women to initiate breastfeeding and to exclusively breastfeed their infants. It is unclear why this should be the case, as the reported rates of breastfeeding initiation in other Middle Eastern countries are similar to those reported for this study, so this association does not reflect necessarily a cultural difference in breastfeeding initiation rates.

A number of biomedical factors were investigated that other studies had shown to be associated with the initiation of breastfeeding. Consistent with other studies of Middle Eastern women in the UAE [46] and Saudi Arabia [37], we found that women who had delivered by caesarean section were less likely to be exclusively breastfeeding at discharge. Newborns are often taken to a nursery following a caesarean section delivery in order to allow the mother to rest after her operation, making it difficult for her to establish breastfeeding and increasing the likelihood of the infant receiving prelacteal and supplementary formula feeds. This negative association has been reported also in a studies of Western [52] and Chinese women [53], and having delivered by caesarean section has been associated with the delayed onset of lactation [54]. We also found that admission of an infant to the SCN was negatively associated with the initiation of breastfeeding and the likelihood of a mother exclusively breastfeeding at discharge from hospital, a finding that has been reported also in studies of Western women [55].

Social support from a woman's partner or other family members has been shown to affect the mother's decision to initiate breastfeeding and we found a significant independent association between the husband's preference for breastfeeding and breastfeeding at discharge. Two studies of women in Saudi Arabia have investigated the influence of paternal attitudes on breastfeeding outcomes, with one finding that mothers were more likely to initiate breastfeeding if their partners supported breastfeeding and encouraged them to initiate exclusive breastfeeding [38], whereas the second study found no association between the Saudi father's attitude towards breastfeeding and breastfeeding initiation [56]. The degree to which a woman's partner will influence her decision to breastfeed varies according to the woman's age, social class and cultural or ethnic background [57]. For instance, Anglo-American women identified their husband as being their major source of support regarding infant feeding decision and less often turned to their mother. On the other hand, women of Latin American origin were more likely to consult their mother on infant feeding matters, although husbands were responsible for most other family decisions [58]. Studies of Muslim women have highlighted the importance of grandmothers both in providing practical support and as major influences on infant feeding decisions [28,43].

There are a number of limitations to this study. Firstly, the sample size is relatively small and this is reflected in the wide confidence intervals around some of the adjusted odds ratios reported. This suggests that more data should be collected before a more definitive statement can be made regarding some of the associations reported here. Secondly, while there was no significant difference in age, level of education and method of feeding between participants and those women who declined to participate, the proportion of women who had undergone a caesarean section in this study is three times that of the national average. The average length of post-partum stay for Kuwaiti public hospitals is a maximum of two nights for uncomplicated deliveries and five nights for a caesarean section. While every attempt was made to recruit mothers within 72 hours, and in most cases 48 hours, of delivery, women who had undergone a caesarean section had a greater chance of being recruited because of their extended hospital stay. Finally, with the data collection methodology employed it was not possible to ascertain the true method of feeding at discharge. Women were surveyed within 72 hours of delivery, so for those women who delivered vaginally the method of feeding at the time of completing the survey is likely to be the same as the method at discharge, but for women who had delivered by caesarean section it probably reflects the feeding method 48 hours prior to discharge and hence may have been subject to change. However, data collected in the six week follow-up survey (not reported here) confirmed that 97% of women who were breastfeeding at the time of completing the baseline questionnaire left hospital breastfeeding, indicating that this was a reliable definition of breastfeeding at discharge. The remaining 3% of women identified as breastfeeding at discharge were lost to follow-up and we were unable to confirm at the six week interview if they actually left hospital breastfeeding. Despite these limitations, this is the first breastfeeding study in Kuwait in recent years and the results reported here are generally consistent with the findings of other studies of Middle Eastern women and/or Western women and can be used to inform future breastfeeding promotion interventions in Kuwait.

## Conclusion

The initiation of breastfeeding is almost universal amongst women in Kuwait however, few women fully breastfeed their infants. The reasons for the high use of prelacteal and supplementary formula feeds warrant further investigation. Hospital policies and staff training are needed to help mothers initiate breastfeeding within a half-hour of birth and to discourage the early introduction and unnecessary use of infant formula in hospital, in order to support the establishment of exclusive breastfeeding among mothers in Kuwait. Governmental health services need to emphasize and support the importance of regular training programs to all hospital staff, especially those involved in antenatal clinics and maternity wards, as they can influence the early infant feeding practices among the new generation of mothers. As a first step, a Health Ministry policy mandating that all government funded hospitals follow the 10 Steps to Successful Breastfeeding and attain Baby Friendly Hospital Initiative accreditation would do much to promote and establish successful exclusive breastfeeding amongst Kuwaiti women.

## Competing interests

The authors have no competing interests to declare.

## Authors' contributions

MD participated in the design of the study, collected the data, performed the statistical analysis and wrote the first draft of the manuscript. JAS conceived of the study, assisted with statistical analysis and helped draft the manuscript. CAE advised on the statistical analysis and commented on drafts of the manuscript and MAS provided assistance with the coordination of the study and commented on drafts of the manuscript. All authors read and approved the final manuscript.
